# Patient-reported healthcare expectations in inflammatory bowel diseases

**DOI:** 10.1371/journal.pone.0197351

**Published:** 2018-05-17

**Authors:** Valérie Pittet, Carla Vaucher, Florian Froehlich, Michel H. Maillard, Pierre Michetti, on behalf of the Swiss IBD Cohort Study Group

**Affiliations:** 1 Institute of Social & Preventive Medicine (IUMSP), Lausanne University Hospital, Lausanne, Switzerland; 2 Division of Gastroenterology & Hepatology, Lausanne University Hospital, Lausanne, Switzerland; 3 Division of Gastroenterology & Hepatology, Basel University Hospital, Basel, Switzerland; 4 Crohn and Colitis Center, Gastroentérologie Beaulieu SA, Lausanne, Switzerland; University Hospital Llandough, UNITED KINGDOM

## Abstract

**Background:**

Patient-reported experience is an important component of a holistic approach to quality of care. Patients’ expectations of treatments and global disease management may indicate their illness representations and their satisfaction and hopes regarding quality of care.

**Objective:**

To study expectations of patients with inflammatory bowel disease.

**Methods:**

Two focus groups were conducted with 14 patients to explore their expectations about treatments and disease management. From qualitative content analyses of focus group discussions, we built a 22-item expectations questionnaire that was sent to 1756 patients of the Swiss IBD cohort. Answers were collected on a visual analog scale from 0 to 100, and medians (interquartile range [IQR]) calculated. Factor analysis identified main expectation dimensions, and multivariate analyses were performed to describe associations with patient characteristics.

**Results:**

Of 1094 patients (62%) included in the study, 54% were female, 54% had Crohn’s disease, 35% had tertiary education, and 72% were employed. Expectation dimensions comprised realistic, predictive, and ideal expectations and were linked to information, communication, daily care, and disease recognition. Half (11 of 22) of the expectations were ranked as very high (median score > 70), the 2 most important being good coordination between general practitioners and specialists (median score: 89, IQR: 71–96) and information on treatment adverse events (89, IQR: 71–96). Women had overall higher levels of expectations than did men. Expectations were not associated with psychosocial measures, except those related to disease recognition, and most of them were highly associated with increased concerns on disease constraints and uncertainty.

**Conclusions:**

Patients have high expectations for information and communication among caregivers, the levels varying by gender and region. Patients also appear to request more active participation in their disease management.

## Introduction

Patients’ expectations play an important role in their assessment of the quality[1,2] and delivery of health services.[3] Healthcare expectations may be defined as anticipated beliefs and values,[4] formed through cognitive processes,[5] related to healthcare processes, events, or outcomes.[6,7] In other words, these expectations may correspond to the difference between expected and experienced healthcare. Assessing healthcare expectations may be a first step in understanding satisfaction with healthcare, although expectation is a much broader concept. Indeed, expectations are multidimensional and complex, they can include passive and active components,[8] and no standard instrument for their evaluation is available.[2] Expectations may be general, related to, for example, access to information, discussions of problems,[9] or psychosocial support, or more specific, related to, for example, requests for specific tests or treatments, coping strategies, and ways to return to “normal” life status, including prevention tips.[10]

Inflammatory bowel disease (IBD) includes Crohn’s disease (CD) and ulcerative colitis (UC), 2 related, chronic, intermittent diseases that cause progressive bowel damage and require lifelong management. Little work has been done, however, to measure patient-reported experience in IBD. Several recent studies assessed the satisfaction of patients who have IBD or their healthcare providers with healthcare services,[11–19] but no study has assessed healthcare expectations in large groups of patients. Satisfaction was much more often assessed because validated quantitative scales exist[19], as compared to expectations that mostly require qualitative exploration and study designs[20]. Involvement of patients with IBD in their own care and their request for more active participation in disease management is a serious topic nowadays, but a broad assessment of how and where patients expect to contribute is lacking. We may indeed ask whether patients who express wide-ranging specific expectations and communicate them regularly to their physician, thus taking the role of active participants in their own care, influence both the cost and quality of care. This may be of particular importance in decision making when the patient has specific requests, considerations, desires, or values that have to be taken into account. Thus, a constructive partnership for better care at a reasonable cost might be formed between knowledgeable, empowered patients and healthcare professionals. For this reason, there is a need to develop knowledge transfer and activation programs for patients with IBD, and to increase direct collaboration with them, in order to improve follow-up and outcomes. We took the opportunity of two ongoing research projects to address the question of what are current expectations of IBD patients regarding their care and treatments: 1) the Swiss IBD national cohort (SIBDC) study starting in 2006[21], and 2) a research project focused on patients and physicians perceptions of appropriateness of care in IBD[22]. In this last project, we used combined methodological study designs to qualitatively explore and quantitatively describe perspectives and possible values of patients regarding risk of benefits of their treatments, and overall care related to their disease. In this purpose, we collect information on concerns[23] and expectations, the last being the focus of the present manuscript.

The aim of this study was, first, to conduct a qualitative study to identify a set of disease and treatment-related expectations of patients with IBD. Second, we performed a cross-sectional study among a large number of IBD patients to quantify the identified expectations and study associated factors.

## Methods

### Study design

We undertook a mixed-method study by using an exploratory sequential design. We first conducted 2 focus group discussions, one with 6 patients with CD and the other with 8 patients with UC, to explore disease- and treatment-related expectations. Patients were selected through the SIBDC. Inclusion criteria were the following: 1) living in the French-speaking region of Switzerland, 2) aged 18 to 70, 3) speaking and understanding French, 4) having been followed regularly by a gastroenterologist for the past 18 months, 5) having been diagnosed with UC or CD for at least 5 years, and 6) having experienced at least 2 different types of treatments during the course of their disease. This study is part of a broader project centered on patient advice on the appropriateness of care in IBD, for which detailed patient selection criteria were used, as previously described.[22] We were interested in investigating the quality of care, more specifically, physicians’ and patients’ perceptions of appropriateness of care and treatments. We thus attempted to collect information related to patients’ values that could be taken into consideration to construct a holistic model of quality of care. Expectations were explored as one of those potential values. In the focus groups, discussions were focused on prioritization and specification of outcomes and processes related to good outcomes among patients who had long-term experience with treatments and disease.[22] We did not discuss situations related to a medical visit and thus did not attempt to assess specific pre-visit expectations, as was done in previous studies.[24,25] In contrast, we were interested in investigating expectations linked to the overall context in which patients evolved in relation to their disease, whether it involved direct contact with physicians or not (ie, having an active disease or being in remission for a long time). Focus group discussions were audio-recorded and transcribed for research purposes with the participants’ written consent. The detailed description of focus groups methodology, conduction and analysis is available in our previous manuscript.[22]. The content of the discussions was analyzed to identify the main categories of expectations addressed.

In a second step, we built a 22-item questionnaire to conduct a cross-sectional survey to explore the prevalence and degree of similarity of expectations of a larger population of patients. Answers to questions (eg, “In relation to the disease I am suffering from, I would need and/or expect…”) were collected on a visual analog scale ranging from 0 to 100 (0 = not at all, 100 = a great deal). Patients could also describe some expectations in more detail in a free text section at the end of the questionnaire. The questionnaires were sent to all adult patients undergoing active follow-up who were enrolled in the Swiss IBD cohort by January 2015. The patient characteristics extracted from the SIBDC 10-year longitudinal database were diagnosis (CD, UC, IBD undetermined) and disease duration. Patient self-reported characteristics, collected through paper questionnaires, included gender, age, language for questionnaires (French/German), education level (none or compulsory, secondary education [professional/general], upper secondary education, tertiary education), working status (employed, in training, at home or unemployed, retired or annuitant), current symptoms severity and frequency (visual analog scale ranging from 0 to 100). We also assessed the association between health-related quality-of-life measures and patient expectations dimensions. We used the Short Form-36 (SF-36) questionnaire, divided into 2 subscores: the Physical Component Summary and the Mental Component Summary. The 32-item Inflammatory Bowel Disease Questionnaire (IBDQ) was assessed with 4 subscores (bowel symptoms, systemic symptoms, emotional function, and social function). Mood was assessed with the Hospital Anxiety and Depression Scale (HADS), which was divided into 2 subscales, one assessing depression and the other anxiety.

Finally, we checked the associations between expectations and concern dimensions (socialization and stigmatization, constraints and uncertainty, impact of the disease on body and mind [including symptoms], loss of body control [including sexuality], disease transmission, and long-term impact of the disease). Concerns were assessed in the same IBD sample, and factor analyses that yielded dimensions were described in detail in a previous manuscript.[23]

### Statistical analysis

Descriptive analyses with numbers and percentages were used to characterize the study population. The median (interquartile range [IQR]) was calculated for each expectation because all measures were non-normally distributed. Missing values were replaced by a score of 50, in accordance with the instructions given to patients: “If you have no opinion or are undecided, please put a cross in the middle of the scale.” We used the Wilcoxon rank-sum test to test the distribution differences of the expectations (statistical significance: p-value <0.002, with Bonferroni correction for multiple testing). We conducted a factor analysis with the principal axis factor method to explore the dimensions of the main expectations.[26] Factors with correlation matrix eigenvalues ≥1 were retained. The factor analysis was conducted with all of the individual expectation items; none of them had too low a communality for justifying its exclusion. Varimax rotation with the Kaiser normalization method was performed, and we retained factors with loadings ≥0.35.[27] Cronbach’s alphas were calculated to assess the internal consistency of items in each dimension. For each dimension, we calculated a non-weighted sum score of each item. The first 3 dimensions were normalized by using power transformation, and the fourth was split into 2 categories. We thus conducted 3 multiple linear regressions and 1 logistic regression to assess associations between expectation dimensions and patient characteristics. To separate the effect of anxiety and depression from the effect of QoL measures, we first conducted a linear regression with QoL measures as dependent variables and anxiety and depression scores as explanatory variables. The residuals of these regressions were used in the multiple linear regressions as explanatory variables.

Because crude multiple linear regression coefficients might be difficult to interpret or compare due to power transformations, results were reported with signs and significance of associations only.

Factor analyses were performed with SPSS Statistics 23 (IBM Corp., New York, NY, USA). Descriptive and regression analyses were performed by using STATA statistical software v.14.1 (STATA Corp., College Station, TX, USA).

### Ethics approval

Ethics approval was obtained from the regional Swiss Ethics Committees in which cohort participants were enrolled (Commission d’éthique du Canton de Vaud/Protocol no. 33/06). Ethics approval was obtained to conduct focus groups (Commission d’éthique du Canton de Vaud /Protocol no. 185/13). Written, informed consent was obtained from each patient included in the study. The study protocol conforms to the ethical guidelines of the 1975 Declaration of Helsinki as reflected in a priori approval by the institution’s human research committee.

## Results

### Expectations expressed in focus groups

A total of 71 patients were contacted for focus groups via postal mail: 10 UC patients answered positively, and 8 finally took part in the UC group (3 males, 5 females); 8 CD patients accepted and 6 (2 males, 4 females) finally took part in the CD group discussion. The range of disease duration was 6 to 28 years for UC patients and 10 to 36 years for CD patients. Content analysis of the focus group discussions yielded expectations in the following main categories: information, treatments, daily disease management, medical care and follow-up, and social life and support. Information was expected about overall treatments, from conventional drugs to complementary and alternative medicine (CAM). CAM was most frequently cited by patients with UC, who had all experienced at least 1 CAM during the course of their disease. More information was also expected on extraintestinal manifestations and comorbidities, the impact of stress and emotional factors on disease exacerbations, diet and flare prevention (patients with UC), and the impact of smoking (patients with CD). Patients expressed a major impact of the disease on their life. Many patients expressed expectations related to having increased freedom and responsibility regarding the disease; this was especially true for patients with CD. Although they were grateful to medical care and treatments for helping them in daily life, they still had many expectations related to social support and recognition of their disease by the general population—including relatives, friends, and those in the professional environment—or of healthcare providers.

### Assessment of expectations in the cross-sectional study

#### Characteristics of the sample population

Among the 1756 adult patients who actively participated in the SIBDC follow-up and agreed to regularly receive self-reported questionnaires, 1123 (64%) sent us the questionnaire back. Of these 29 did not fill in the expectation questions, thus 1094 patients (62%) could be included in the present study. Slightly more than two-thirds of the patients lived in the German-speaking part of Switzerland and 31% in the French-speaking part (Table 1).

**Table 1:** Baseline characteristics of patients included in the cross-sectional survey.

Variable	N (%)
All	1094
**German speakers**	755 (69.0)
**Female gender**	595 (54.4)
**Age**	
*≤40 years*	387 (35.4)
*>40 years*	707 (64.6)
**Age at diagnosis**	
*≤40 years*	851 (78.0)
*>40 years*	240 (22.0)
**Disease duration**	
*<5 years*	146 (13.4)
*5–15 years*	485 (44.4)
*>15 years*	460 (42.2)
**Diagnosis**	
*Crohn’s disease*	591 (54.0)
*Ulcerative colitis*	473 (43.2)
*IBD undetermined*	30 (2.7)
**Education level**	
*None or compulsory*	99 (9.6)
*Secondary education (professional)*	407 (39.3)
*Secondary education (general)*	165 (15.9)
*Upper secondary education*	212 (20.5)
*Tertiary education*	152 (14.7)
**Working status**	
*Employed*	737 (71.5)
*In training*	30 (2.9)
*At home/unemployed*	100 (9.7)
*Retired/annuitant*	164 (15.9)

About half of the respondents were women, two-thirds were more than 40 years old, and a large majority (74%) were professionally active or in training. About one-third of all patients had an upper secondary or tertiary education. Half of patients had CD, 78% were diagnosed before 40 years, and 87% had experienced IBD for 5 years or more.

#### Highest individual expectations

**Fig 1:**
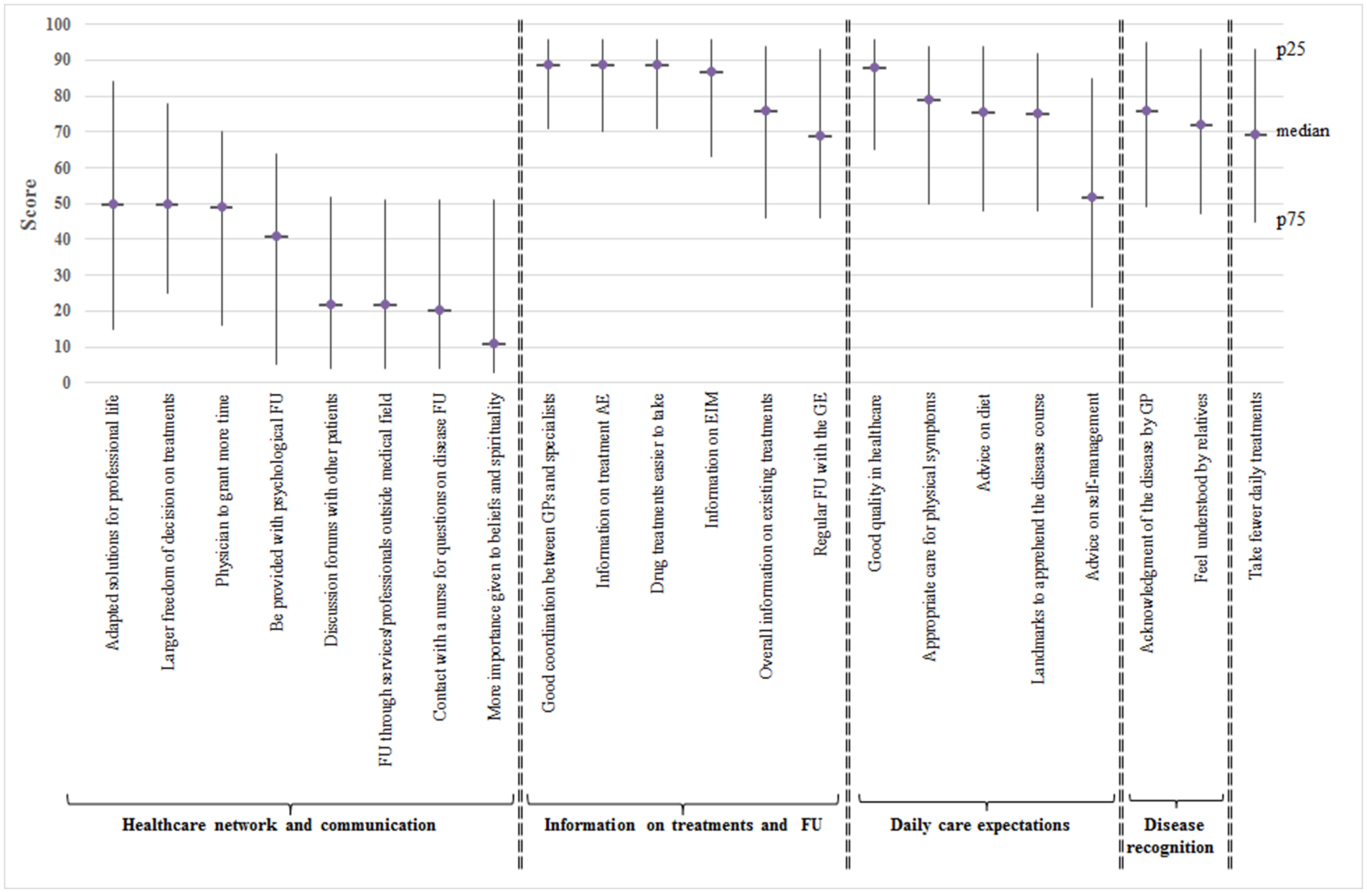
Comparison of median (interquartile range) scores for individual healthcare expectations in the SIBDC patient survey.

Overall, 11 of 22 expectations were ranked as very high, with a median score of over 70. The 5 higher individual expectations (Fig 1) were linked to “good coordination between general practitioners (GPs) and specialists” (median score: 89, IQR: 71–96), “information on treatment adverse events” (median score: 89, IQR: 71–96), “drug treatments easier to take (median score: 89, IQR: 70–96), “good quality in healthcare” (median score: 87, IQR: 66–96), and “information on extraintestinal manifestations” (median score: 80, IQR: 54–95).

#### Main dimensions of expectations

The factor analysis yielded 4 main expectation dimensions, which were labeled according to the item(s) with the strongest factor loading (Table 2): (1) healthcare network and communication, (2) information on treatments and follow-up, (3) daily care expectations, and (4) disease recognition. Cronbach’s alpha, which measures the internal consistency among items included in each dimension, varied between 0.77 and 0.71. All dimensions explained 37.7% of the common variance. One item (“Take fewer daily treatments”) had too low a factor loading and could therefore not be associated with any dimension.

**Table 2:** Results of the rotated factor matrix performed on data from the SIBDC patient survey.

	Healthcare network and communication	Information on treatments and follow-up	Daily care expectations	Disease recognition
FU through services/professionals outside the medical field	0.66			
Contact with nurse for questions on disease FU	0.57			
Be provided with psychological FU	0.52			
Discussion forums with other patients	0.51			
Larger freedom of decision related to treatments	0.48			
More importance given to beliefs and spirituality	0.44			
Physician to grant more time	0.40			
Adapted solutions for professional life	0.38			
Information on treatment AEs		0.67		
Good coordination between GP and specialists		0.62		
Drug treatments easier to take		0.50		
A regular FU with the GE		0.40		
Overall information on existing treatments		0.40		
Information on EIMs		0.37	(0.37)	
Appropriate care for physical symptoms			0.56	(0.41)
Landmarks to apprehend the disease course			0.53	
Advice on self-management	(0.50)		0.46	
Advice on diet			0.42	
Good quality in healthcare			0.39	
Feel understood by relatives				0.69
Acknowledgment of the disease by GP		(0.41)		0.55
*Take fewer daily treatments*				
**% of total variance explained**	**12.9**	**9.8**	**8.3**	**6.7**
**Cronbach’s alpha**	**0.77**	**0.73**	**0.73**	**0.71**

FU = follow-up, AEs = adverse events, GP = general practitioner, GE = gastroenterologist, EIMs = extraintestinal manifestations.

#### Factors associated with expectation dimensions

Healthcare network and communication expectations were significantly higher for women (p = 0.013), Table 3, and decreased with higher level of education. Those expectations were associated with higher concerns related to socialization & stigmatization and disease constraints & uncertainty (p<0.001), and with lower concerns on disease transmission (p = 0.003). Regarding individual items in this dimension, we found that women rated the following significantly higher than did men: having adapted solutions for professional life (median score: 52 vs 50), being provided with psychological follow-up (median score: 45 vs 21), and being followed up with services or professionals outside the medical field (median score: 36 vs 15) (Table 4).

**Table 3:** Results of multivariate regressions for each expectation dimension performed on data from the SIBDC patient survey. Values indicate the sign of the coefficient (direction of the association) and the p-value. Bold indicates significant associations.

	Healthcare network and communication	Information on treatments and follow-up	Daily care expectations	Disease recognition
**French speakers**	+/0.357	**+/0.046**	**+/0.001**	+/0.427
**Female gender**	**+/0.013**	**+/0.002**	**+/0.001**	**+/0.005**
**Age > 40 years**	+/0.120	**+/<0.001**	**+/<0.001**	+/0.989
**Disease duration**				
*<5 years*	Ref	Ref	Ref	Ref
*5–15 years*	-/0.751	+/0.998	+/0.977	-/0.308
*>15 years*	-/0.165	-/0.665	-/0.092	+/0.849
**Diagnosis**				
*Crohn’s disease*	Ref	Ref	Ref	Ref
*Ulcerative colitis*	+/0.199	+/0.796	+/0.313	**+/0.001**
*IBD undetermined*	-/0.543	-/0.959	+/0.907	+/0.641
**Symptoms severity**	+/0.865	+/0.893	+/0.314	+/0.482
**Symptoms frequency**	-/0.552	-/0.169	-/0.292	+/0.574
**Education level**				
*None or compulsory*	Ref	Ref	Ref	Ref
*Secondary education (professional)*	-/0.314	+/0.406	+/0.188	-/0.414
*Secondary education (general)*	**-/0.038**	-/0.850	+/0.580	-/0.540
*Upper secondary education*	**-/0.030**	-/0.409	+/0.303	-/0.061
*Tertiary education*	**-/0.018**	-/0.497	+/0.069	-/0.122
**Working status**				
*Employed*	Ref	Ref	Ref	Ref
*In training*	-/0.496	-/0.234	+/0.317	-/0.307
*At home/unemployed*	-/0.066	**-/0.033**	**-/<0.001**	-/0.107
*Retired/annuitant*	-/0.086	+/0.067	-/0.525	+/0.298
**Signs of anxiety**				
*None*	Ref	Ref	Ref	Ref
*Mild*	+/0.191	+/0.112	-/0.776	-/0.974
*Moderate*	-/0.216	+/0.929	-/0.105	-/0.199
*Severe*	+/0.241	+/0.145	+/0.310	+/0.425
**Signs of depression**				
*None*	Ref	Ref	Ref	Ref
*Mild*	+/0.125	-/0.444	+/0.535	+/0.925
*Moderate*	-/0.517	+/0.966	-/0.202	+/0.439
*Severe*	-/0.188	-/0.725	+/0.819	+/0.985
IBDQ bowel* **subscore**	+/0.074	+/0.069	-/0.649	**+/0.024**
IBDQ systemic* **subscore**	+/0.366	+/0.830	+/0.696	+/0.078
IBDQ emotional* **subscore**	-/0.542	+/0.805	-/0.371	**-/0.026**
IBDQ social* **subscore**	-/0.165	-/0.376	+/0.642	+/0.161
SF-36 Physical Component Summary (PCS)*	-/0.629	-/0.115	+/0.588	**-/0.014**
SF-36 Mental Component Summary (MCS)*	-/0.173	-/0.616	+/0.278	+/0.638
**Concerns main dimensions**				
*Socialization and stigmatization*	**+/<0.001**	-/0.191	+/0.242	+/0.101
*Constraints and uncertainty*	**+/<0.001**	**+/0.004**	**+/<0.001**	-/0.970
*Symptoms (impact on body and mind)*	+/0.534	+/0.347	+/0.069	+/0.173
*Loss of body control (including sexuality)*	-/0.991	**+/0.004**	**+/0.047**	+/0.211
*Disease transmission*	**-/0.003**	-/0.055	-/0.267	-/0.989
*Long-term impact of the disease*	-/0.119	+/0.229	-/0.163	**-/0.023**

* Component unrelated to anxiety and depression. IBDQ: Inflammatory Bowel Disease Questionnaire

**Table 4:** Scores for individual healthcare expectations of SIBDC patients according to gender, disease type, region, and age. Values are medians (interquartile range).

	Gender		Diagnosis		Language		Age	
Dimension	Men	Women	CD	UC	German	French	Age ≤40	Age >40
Good coordination between general practitioners and specialists	**87 (67–96)**	**91 (73–97)**	90 (71–96)	88 (70–96)	88 (71–96)	91 (71–97)	**81 (61–96)**	**91 (77–96)**
Information on treatment adverse effects	87 (69–96)	90 (74–96)	89 (71–96)	89 (71–96)	88 (70–96)	90 (76–97)	**85 (64–96)**	**90 (76–96)**
Drug treatments easier to take	88 (65–96)	90 (74–97)	88 (66–96)	90 (74–96)	**87 (64–96)**	**92 (80–97)**	85 (65–96)	90 (76–96)
Good quality in healthcare	87 (66–96)	89 (63–97)	90 (67–96)	87 (64–96)	87 (67–96)	91 (59–97)	84 (67–96)	90 (63–96)
Information on extraintestinal manifestations	**80 (54–95)**	**90 (70–97)**	87 (63–96)	86 (64–96)	85 (63–96)	89 (65–97)	79 (56–96)	89 (66–96)
Appropriate care for physical symptoms	**72 (49–90)**	**84 (52–95)**	80 (50–94)	78 (50–93)	76 (49–93)	84 (53–95)	75 (50–92)	81 (50–94)
Acknowledgment of the disease by general practitioners	**67 (48–93)**	**80 (49–96)**	76 (48–95)	75 (49–94)	72 (48–94)	82 (50–95)	73 (48–94)	78 (49–95)
Overall information on existing treatments	73 (46–93)	81 (47–95)	75 (46–94)	78 (48–95)	**72 (44–93)**	**86 (53–96)**	72 (44–92)	79 (47–95)
Advice on diet	**70 (47–92)**	**82 (49–95)**	72 (47–94)	79 (50–94)	74 (48–94)	79 (48–95)	74 (47–94)	77 (48–94)
Landmarks to apprehend the disease course	74 (49–91)	76 (47–93)	73 (47–92)	76 (49–93)	74 (48–92)	76 (48–93)	74 (49–90)	75 (48–93)
Feel understood by relatives	**60 (42–90)**	**78 (49–95)**	73 (48–94)	72 (47–92)	73 (48–93)	71 (44–94)	73 (49–93)	71 (46–94)
Take fewer daily treatments	68 (41–92)	72 (46–94)	59 (38–93)	76 (48–94)	**74 (47–94)**	**55 (22–92)**	68 (46–94)	71 (44–93)
Regular follow-up with the gastroenterologist	65 (43–92)	72 (47–94)	68 (46–93)	70 (46–93)	**63 (44–91)**	**79 (49–95)**	**57 (39–84)**	**76 (48–94)**
Advice on self-management	**50 (16–77)**	**54 (32–90)**	52 (18–83)	54 (32–88)	**50 (16–76)**	**74 (46–93)**	51 (24–79)	53 (18–89)
Adapted solutions for professional life	**50 (10–77)**	**52 (22–89)**	51 (18–88)	50 (14–80)	50 (16–82)	50 (11–89)	53 (20–86)	50 (12–83)
Larger freedom of decision on treatments	50 (23–72)	51 (25–83)	51 (26–80)	50 (24–77)	50 (27–76)	51 (16–83)	50 (30–77)	50 (20–79)
Physician to grant more time	49 (17–69)	49 (15–73)	48 (16–67)	49 (17–75)	49 (16–73)	48 (16–58)	49 (25–71)	49 (12–70)
Be provided with psychological follow-up	**21 (5–53)**	**45 (6–76)**	33 (5–60)	44 (6–66)	41 (5–63)	29 (5–65)	44 (6–73)	30 (5–58)
Discussion forums with other patients	21 (4–50)	22 (4–55)	22 (4–53)	22 (4–51)	22 (4–51)	21 (4–56)	21 (4–51)	23 (4–53)
Follow-up through services or professionals outside the medical field	**15 (4–50)**	**36 (5–53)**	20 (4–51)	26 (5–52)	**17 (4–49)**	**45 (5–72)**	25 (5–51)	20 (4–52)
Contact a nurse for questions on disease follow-up	17 (4–50)	22 (3–52)	18 (4–51)	22 (4–51)	**17 (3–49)**	**43 (4–69)**	22 (3–50)	19 (4–52)
More importance given to beliefs and spirituality	9 (2–50)	16 (3–52)	10 (3–52)	14 (3–51)	13 (3–51)	8 (2–51)	9 (2–51)	13 (3–51)

Bold corresponds to a p-value of <0.002 (statistically significant distribution differences).

By analyzing patients’ comments (free text section at the end of the questionnaire), we found that specific expectations were also related to CAM. Indeed, patients asked for better coordination between CAM and conventional treatments, more openness about CAM from gastroenterologists, more information about CAM, and CAM reimbursement. We also observed differences in items in the healthcare network and communication dimension according to region. Indeed, patients from the French-speaking part of Switzerland had higher expectations related to services or professionals outside the medical field (median score: 45) and to potential contact with a nurse for questions about follow-up (median score: 43) than did those from the German-speaking part (median score: 17 for both), although regional differences were not perceptible by looking at the dimension as a whole.

Women, French speakers, and patients more than 40 years old had significantly higher expectations related to information on treatments and follow-up than did men (p = 0.002), German speakers (p = 0.046), and younger patients (p<0.001). Those expectations were lower among patients at home or unemployed, as compared to employed patients (p = 0.033). Expectations related to information on treatments and follow-up were associated to higher concerns on disease constraints & uncertainty and on loss of body control (including sexuality) (p = 0.004). Patients over 40 years more frequently expected good coordination between GPs and specialists, information on treatment adverse events, or regular follow-up with the gastroenterologist (median scores: 91, 90, and 76, respectively) than did younger patients (median scores: 81, 85 and 57, respectively). Women had higher expectations related to coordination between GPs and specialists (median score: 91) and related to extraintestinal symptoms and manifestations (median score: 90) than did men (median scores: 87 and 80, respectively). French speakers more frequently expected drug treatments that would be easier to take, overall information on existing treatments, and regular follow-up with the gastroenterologist (median scores: 92, 86, and 87, respectively) than did patients from the German-speaking part of Switzerland (median scores: 79, 72, and 63, respectively). Regarding treatments, some patients expected more information, not only about medications, but also about the cessation of these treatments. Good coordination between GPs and specialists was also related to IBD and other comorbidities because of the need to take concomitant treatments and the potential risk of drug interactions.

Daily care expectations were significantly higher among French speakers (p = 0.001), women (p = 0.001) and patients aged more than 40 (p<0.001), and were lower among patients at home or unemployed, as compared to employed patients (p<0.001). Daily care expectations were associated with higher concerns related to constraints & uncertainty (p<0.001) and loss of body control (p = 0.047). Women had higher expectation scores regarding receiving appropriate care for physical symptoms and advice on diet and on self-management (median scores: 84, 82, and 54, respectively) than did men (median scores: 72, 70, and 50, respectively). French speakers had higher expectations related to self-management than did German speakers (median scores: 74 vs 50). Specific expectations were also expressed by patients about knowing more about the influence of sport activities on the disease, having more information on nutrition, and receiving dietary recommendations.

Women and UC patients reported higher expectations related to disease recognition than did men (p = 0.005) and patients with CD (p = 0.001), those expectations being related to GPs (median scores: 80 vs 67) or relatives (median scores: 78 vs 60). Detailed needs related to this topic were expressed as follows: recognition of the disease and its symptoms by the physician; recognition of the disease in the general population (eg, priority lanes), by social insurance (eg, disability, complications), and in the professional environment (eg, for better stress management, working load-agenda adaptations); recognition of the influence of stress on the disease; and recognition of the chronicity of the disease. Expectations related to disease recognition were associated with lower SF-36 physical QoL (p = 0.014), lower IBDQ emotional subscore (p = 0.026), and higher IBDQ bowel subscore (p = 0.024). These expectations were associated with lower concerns related to the long-term impact of the disease (p = 0.023).

## Discussion

In this study, we aimed to explore, quantify, and describe the expectations of patients with IBD related to their disease and treatments. Expectations were first explored through a qualitative study based on 2 focus group discussions. A set of 22 expectations derived from content analyses of these discussions was used to survey patients with IBD who were included in a nationwide cohort study. We found 4 main expectation dimensions related to network and communication, information, daily care, and disease recognition. Two-thirds of all expectations were given a median score of over 50 and one-quarter a median score of over 87, which was very high. Women had significantly higher expectation levels than did men, regardless of the dimension. Expectations were not associated with psychosocial measures, except those related to disease recognition, and most of them were highly associated with increased concerns on disease constraints and uncertainty.

This is the first study that aimed to identify patient-reported expectations about disease and treatments in a large set of patients with IBD. Therefore, an overall comparison with previous similar studies is not possible. In relation to previous theories and studies on expectations, we could categorize the main dimensions of expectations as follows: healthcare network and communication, as well as information on treatments and follow-up, might both be considered realistic expectations, the first being active behavior or activation of the patient and the second more passive behavior. Daily care expectations are predicted expectations[6] in that they may reflect what the patients expect to be beneficial for improving their outcomes. Finally, disease recognition expectations are ideal or value expectations, related to hopes and desire for a better life[3] and social integration. As mentioned by Bowling et al,[2] expectations are complex to describe and understand. We found few independent variables associated with expectations. Symptoms severity or frequency were not associated with expectations, and, globally, expectations did not vary according to diagnosis. We found that women concerned about disease constraints and uncertainty, i.e. reflecting the chronicity of the disease and its associated treatments and outcomes management, are those with higher information and communication expectations. We assessed expectations levels, not satisfaction with healthcare. Therefore, we could not further explore whether this only reflects a gender perspective (i.e., different levels of information and communication expectations but equal levels of satisfaction), or if there is an actual variation in the IBD healthcare management according to gender leading to increased or unmet expectations.

Some expectations were particular (e.g., “take fewer daily treatments”), others more general (e.g., “Good quality of healthcare”), which reflect the way patients expressed them during focus groups. This might indicate that general expectations like good quality of healthcare, for which the answer is not straightforward, would probably benefit to be further and individually explored to break down all related aspects, depending also on healthcare system were patients may evolve. As an example, one recent publication was performed in Sweden, exactly focused on this question of exploring patients’ perceptions of healthcare, indicating that expectations might be eg. in direction of respectful and trustful relationship, facilitating healthcare staff and patients to work as a team in fulfilling individual needs[20].

Expectations linked to information were highly expressed by patients, as shown in previous studies. Casellas et al[16] showed that satisfaction with information was scored the lowest of the 6 rated domains and that lack of information was a constant complaint of patients over time, especially for those with mild to moderate conditions who were not mainly followed by gastroenterologists.[28] Some patients’ views of quality improvements in gastrointestinal diseases were directed at increasing access to patient organizations and groups, having consistency and coordination between GPs and hospital management,[29] improving knowledge of GPs on IBD,[28,30] and being more involved in defining disease-related concerns and prioritization of outcomes.[29] We also observed that communication within the healthcare network was not considered optimal. This finding may indicate that knowledge transfer tools need to be developed urgently, not only from physicians to patients, but also within the physicians’ community, especially for primary care physicians, who did not appear to be always up to date with IBD care or treatments.[31,32] On the basis of observations made within groups of GPs[33] and the report of their attitudes, their lack of knowledge, and the difficulties they face with the management of IBD care and treatments, healthcare initiatives should probably go towards establishing the use of a chronic care model approach. Such attempts have been questioned and tested recently in Australia and the United States[34–37] and could be used not only by integrating GPs, but also by integrating psychological advice or follow-up.

Our findings also showed that patients had a number of daily care expectations that may be interpreted as a willingness to participate more actively in the decisions regarding treatments, potential prevention, and anticipation of flares. The desire of patients to be actors in their own follow-up and the willingness of gastroenterologists to take this into account is not new.[38] Attempts have been made to increase shared decision making[39] and to build patient activation programs,[40,41] but this appears to be still in its early stages and more difficult to develop than expected, at least in Switzerland. Despite the ongoing longitudinal data collection in cohort studies or registries, integrated solutions offering optimal on-site decision-sharing tools, where doctors and patients can equally contribute and access information, are lacking. Interesting projects in the direction of patient empowerment have recently emerged,[42] in parallel with increasing attention given to assessing patients’ requirements and their need to access electronic health records or personal health records, as well as to the potential contribution to chronic disease management.[43] Interestingly, daily care expectations were significantly higher among French-speakers as compared to German-speakers, which indicates that cultural sensitivity is an important issue to be considered, at least in Switzerland. French-speakers patients had higher levels of expectations as German-speakers regarding follow-up management (i.e., regular follow-up with gastroenterologist, contact with a nurse, advices on self-management and follow-up through services or professionals outside the medical field). This may also indicate that generalizing expectations at a global population level is difficult, unless they are stratified for specific targeted groups. Based on this observation, we could e.g., argue that groups of patients from all major linguistic regions should be equally involved, when developing patient empowerment tools expected to be used at a country level.

Finally, we observed that disease recognition expectations were given very high scores. This is in line with patients’ concerns about these issues,[23] as well as observations from recent studies indicating that stigmatization in gastrointestinal diseases, especially IBD, is still present.[44] Indeed, although patients are perhaps more prone or willing to share their experiences with the disease now than they were in the past, IBD does not seem to be recognized or accepted in all social circles, especially for women and CD patients. Patients with disease recognition expectations had less concerns on the long-term impact of the disease, which might reflect they have more concerns on the short-term, i.e., the current impact of the disease.

The main strength of our study is related to the large sample size of patients with IBD who could be surveyed. We could investigate patients’ expectations at a national level, with patients followed in university centers, regional hospitals, or private practices. Our goal was to describe patients’ expectations, not to validate a psychometric tool, while acknowledging that expectations might evolve with time. Moreover, we did not aim to measure satisfaction with healthcare. One limitation is related to the survey’s response rate, which may lead to a nonresponse bias, although the impact on the results, in terms of potentially different distributions of expectation ratings among non-respondents, remains undetermined. Another limitation may be related to our results being more representative of patients with long-standing disease than newly diagnosed. Indeed, the large majority of patients who accepted to participate to focus groups, as well as responders to the questionnaire, had a disease duration of 5 years and over. It thus seem to be more difficult to capture expectations of patients diagnosed for a short time. We could however hypothesize that expectations may be, at that time, more difficult to express for those having only a short experience of their disease, and especially less experience with the chronic aspect of the disease. We consider our work as a first attempt to capture patients’ expectations, but a deeper qualitative assessment of expectations is probably needed for groups of newly diagnosed versus patients with long-standing disease to get more insight in the whole spectrum of patients’ expectations.

In conclusion, this study showed patients have high expectations for information and communication among caregivers, the levels varying by gender and region. Patients also appear to request more active participation in their disease management, which is an important signal, first because we could benefit from potential newly generated patient data data to improve or validate, eg. patient-reported outcomes, second because it is a step towards the development of a chronic care model where the patient could contribute.
